# Supplementary material for: The proteasome 19S cap and its ubiquitin receptors provide a versatile recognition platform for substrates
Source: Nat Commun. 2020 Jan 24;11:477. doi: 10.1038/s41467-019-13906-8 (PMC6981147; doi:10.1038/s41467-019-13906-8)
Supplement: Supplementary file 1 — Supplementary Information [file 41467_2019_13906_MOESM1_ESM.pdf]

Supplementary Information

The Proteasome 19S Cap and its Ubiquitin Receptors Provide a Versatile Recognition Platform for Substrates

K. Martinez-Fonts et al.

Supplementary Note 1

We tested several proteasome reconstitution ratios: 1:1, 1:2, and 1:3 CP:RP. The 1:1 reconstitution ratio left substantial free core particle and degraded substrates with the slowest rate (Figure S2A and B). The 1:2 and 1:3 reconstitutions had no detectable free core particle but the 1:3 reconstitution resulted in almost complete conversion to doubly capped proteasome. Both of these reconstitutions degraded a model substrate at similar rates. Free RP would compete for substrate binding and, therefore, we used the 1:2 reconstitution in all future experiments (Figure S2A and B). The reconstituted proteasomes degrade the substrate at a slower initial rate than proteasome purified through its FLAG-tagged RP in the absence of NaCl (holoproteasome) (not shown). This difference could be the result of the loss of proteasome-associated proteins or a direct effect of the high salt wash on proteasome activity.

Supplementary Table 1: Yeast Strains Used in this Paper

Strain name	Genotype	Proteasome label
SY1960a	*MAT α RPN11-FLAG::HIS rpn1-AKAA-ARR::HGR rpn10-uim::KANmx rpn13-pru::NATmx UBP6-pADH1-Ub::URAmx*	QM (quadruple mutant)
SY1568b	*MAT α RPN11-FLAG::HIS3 rpn1-ARR::TRP1 rpn10-uim::KANmx rpn13-pru::NATmx*	TM (triple mutant)
SY1569b	*MAT α RPN11-FLAG::HIS3 rpn1-ARR::TRP1 rpn10-uim::KANmx*	Rpn13
SY1570b	*MAT α RPN11-FLAG::HIS3 rpn1-ARR::TRP1 rpn13-pru::NATmx*	Rpn10
SY1571b	*MAT α RPN11-FLAG::HIS3 rpn1-ARR::TRP1*	Rpn10/13
SY1572b	*MAT α RPN11-FLAG::HIS3 rpn10-uim::KANmx rpn13-pru::NATmx*	Rpn1
SY1574b	*MAT α RPN11-FLAG::HIS3 rpn13-pru::NATmx*	Rpn1/10
YKMF01b	*MAT α RPN11-FLAG::HIS3 rpn1-ARR::TRP1 rpn10-uim::KANmx rpn13::URA3*	TM Δ13
YKMF02b	*MAT α RPN11-FLAG::HIS3 rpn1-ARR::TRP1 rpn13::URA3*	Rpn10 Δ13
YY40 *	*MAT a RPN11-FLAG::HIS3*	Wildtype (WT)
YY37 *	*MAT a PRE1-FLAG::HIS3*	CP

*Strains published in ref.[1]. All other strains were generated for this study and are isogenic to SUB61 (*MAT α lys2-801 leu2-3,2-112 ura3-52 his3- Δ200 trp1-1(am)* ref.[2].

The *rpn10-uim* allele carries the mutations (L228N, A229N, M230N, A231N, and L232N) ref.[3]. The *rpn13-pru* allele carries the mutations E41K, E42K, L43A, F45A, and S93D ref.[4]. The *rpn1-ARR* allele carries the mutations D541A, D548R, and E552R ref.[4]. The rpn1-AKAA-ARR allele carries T1 site mutations (D541A, D548R, and E552R) as well as T2 site mutations (L430A D431K Q434A Q435A) ref.[4]. Mutation of the T2 site is expected to result in ubiquitin deficiency caused by a loss of Ubp6 association. Strain SY1960a carries an additional ubiquitin gene driven by the strong ADH1 promoter and integrated at the UBP6 locus to compensate.

Supplementary Table 2: Initial rates of degradation for the indicated substrates (rows) by the indicated proteasome particles

	WT	Rpn10	Rpn13	Rpn1	TM	Rpn1/10	Rpn10/13	TMΔ13
Ub_5(K48)-GFP-35	0.84±0.05	0.78±0.04	0.05±0.00	0.04±0.01	0.03±0.01			
Ub_5(K48)-GFP-95	1.02±0.05	0.79±0.04	0.08±0.00	0.09±0.00	0.05±0.00			
Ub_9(K48)-GFP-35	0.76±0.04	0.62±0.04	0.07±0.00	0.06±0.01	0.03±0.00			
95-GFP-Ub_5(K48)	0.12±0.01	0.12±0.01	0.00±0.04	0.00±0.00	0.00±0.09			
Ub_5(K63)-GFP-35	0.21±0.01	0.11±0.01	0.06±0.01	0.01±0.01	0.01±0.01	0.21±0.01	0.18±0.08	
Ub_5(K63)-GFP-95	0.36±0.02	0.18±0.01	0.09±0.01	0.06±0.01	0.02±0.00	0.29±0.02	0.25±0.02	
Ub_9(K63)-GFP-95	0.21±0.01	0.13±0.01	0.06±0.00	0.04±0.01	0.01±0.00	0.14±0.01	0.16±0.01	
95-GFP-Ub_5(K63)	0.67±0.08	0.17±0.02	0.02±0.01	0.2±0.2	-0.01±0.01	0.23±0.03	0.30±0.06	
35-GFP-Ub_5(K63)	0.22±0.00	0.14±0.00	0.02±0.01	0.01±0.06	0.0±0.1	0.23±0.01	0.13±0.00	
Ub-35-Ub-GFP-35	0.30±0.02	0.15±0.01	0.05±0.01	0.03±0.01	0.01±0.01	0.36±0.03	0.10±0.02	
Ub_3-35-Ub_3-GFP-35	0.64±0.02	0.73±0.03	0.39±0.02	0.34±0.01	0.12±0.01			
UBL-GFP-35	0.08±0.00	0.20±0.01	0.27±0.00	0.01±0.02	0.01±0.02			0.21±0.01
UBL-GFP-95	0.43±0.02	0.20±0.01	0.49±0.02	0.31±0.01	0.14±0.01			0.18±0.01
Ub_4(lin)-GFP-35	0.27±0.01	0.16±0.00	0.07±0.00	0.02±0.01	0.02±0.00			

Initial rates are given in nM/min; errors are standard errors derived from curve fitting of at least three independent experiments.

Supplementary Table 3: Inhibition constants (K_i) in μM for the indicated ubiquitin chains

Substrate	Proteasome	Competitor					
		Ub_8(K48)	Ub_4(K48)	Ub_4(K63)	Ub_4(K11)	Ub_4(lin)	Ub_4(M1)
Ub_5(K48)-GFP-35	WT	0.21±0.02	1.00±0.09	4.4±0.6	5±1	1.8±0.3	11±3
Ub_5(K48)-GFP-35	Rpn10	0.37±0.05	1.2±0.3	9±1	5.0±0.9	8±1	295±209
Ub_4(lin)-GFP-35	Rpn13	21±1	160±68	5.4±0.8	7±2	2.0±0.5	40±16

Inhibition constants (K_i) are giving in μM; errors are standard errors derived from curve fitting of at least three independent experiments.

Supplementary Table 4: Values of Michaelis-Menten Analysis of Ubiquitinated Substrates

Substrate	Proteasome	K_M nM	V_{max} nM min^{-1}	k_{cat} min^{-1}	k_{cat}/K_M min^{-1} μM^{-1}
Ub_5(K48)-GFP-35	WT	105±14	16.1±0.6	0.65±0.02	6.2±0.8
	Rpn10	155±11	40.2±0.9	1.61±0.04	10.4±0.7
	Rpn13	5,718±809	22±3	0.9±0.1	0.2±0.01
	TM	5,282±771	20±3	0.8±0.1	0.2±0.01
	Rpn1/10	145±10	38.1±0.8	1.52±0.03	10.5±0. 7
	Rpn10/13	58±8	13.0±0.5	0.52±0.02	9±1
	Rpn10 Δ13	103±4	39.5±0.4	1.58±0.02	15.3±0.5
Ub_9(K48)-GFP-35	WT	50±9	2±0.4	0.37±0.02	7±1
	Rpn10	104±10	22.5±0.7	0.90±0.03	8.7±0.8
	Rpn13	1,100±120	7.4±0.5	0.30±0.02	0.3±0.02
	TM	1,678±318	7±1	0.28±0.04	2±0.02
Ub_5(K63)-GFP-35	WT	333±28	11.9±0.4	0.48±0.02	1.4±0.1
	Rpn10	428±14	17.4±0.3	0.70±0.01	1.6±0.04
	Rpn13	816±108	7.7±0.5	0.31±0.02	0.4±0.04
	TM	2,062±461	3.8±0.6	0.15±0.03	0.1±0.01

Errors are standard errors derived from curve fitting of at least three independent experiments.

Supplementary Table 5: Primers used organized by construct name

Construct	Primer Name	Primer number	Primer sequence
35-CP8-Ub	BamHI-35ΔK F	oKMF 083	ATCGTCAGGATCCGCTAAGATACCAACCTTTACTAAGAATCTCG
35-CP8-Ub	35ΔK-EcoRI R	oKMF 084	ATCGTGATCGAATTCTTCAGCGGGCGAAATCCTACCGCTGAT
95-CP8-Ub	BamHI-35ΔK F	oKMF 083	ATCGTCAGGATCCGCTAAGATACCAACCTTTACTAAGAATCTCG
95-CP8-Ub	95ΔK-EcoRI R	oKMF 098	ATGTCTACTGAATTCTTCAGCGGGCGAAATCCTTTGTCTATTC
Ub_5(M1)-CP8-35	SacI-Ubi4 F	oKMF 051	ATCTGCTGAGCTCATGCAGATTTTCGTCAAGACTTTGAC
Ub_5(M1)-CP8-35	Ubi4-BamHI R	oKMF 053	TCGTACTGGGATCCTCAGTTACCACCCCTCAACCTCAAGACAAGG
Ub-35-Ub-CP8-35	Ub2 F	oKMF 094	ATGCAGATATTTGTGAAGACTTTGACCGGT
Ub-35-Ub-CP8-35	Ub2 R	oKMF 095	AACACCACGCAGACGCAGAACCAG
Ub-35-Ub-CP8-35	Ub-35ΔK IF F	oKMF 096	CTGCGTCTGCGTGGTGTTAGATACCAACCTTTACTAAGAATCTCGCAGAACTGTGAG
Ub-35-Ub-CP8-35	35ΔK-Ub IF R	oKMF 097	CTTCACAAATATCTGCATTTCAGCGGGCGAAATCCTACCGCTGATCGT
UbK48R	Ub C/K48R QC F	oKMF 075	GATCAACAAAGATTGATCTTTGCCGGTCGCCAGCTAGAAGACGGTAGAACGCTGTCCGAT
UbK48R	Ub C/K48R QC R	oKMF 076	ATCGGACAGCGTTCTACCGTCTTCTAGCTGGCGACCGGCAAAGATCAATCTTTGTTGATC
UbK63R	Ub-K63R QC F	oKMF 073	ACGCTGTCCGATTACAACATTCAGCGCGAGTCCACCTTACATCTTGTG
UbK63R	Ub-K63R QC R	oKMF 074	CACAAGATGTAAGGTGGACTCGCGCTGAATGTTGTAATCGGACAGCGT
His_6-pp-UbK11R	Ub-K11R QC F	oKMF 073	ACGCTGTCCGATTACAACATTCAGCGCGAGTCCACCTTACATCTTGTG
His_6-pp-UbK11R	Ub-K11R QC R	oKMF 074	CACAAGATGTAAGGTGGACTCGCGCTGAATGTTGTAATCGGACAGCGT
His_6-pp-UbK48R	Ub C/K48R QC F	oKMF 075	GATCAACAAAGATTGATCTTTGCCGGTCGCCAGCTAGAAGACGGTAGAACGCTGTCCGAT
His_6-pp-UbK48R	Ub C/K48R QC R	oKMF 076	ATCGGACAGCGTTCTACCGTCTTCTAGCTGGCGACCGGCAAAGATCAATCTTTGTTGATC
His_6-pp-UbK63R	Ub-K63R QC F	oKMF 073	ACGCTGTCCGATTACAACATTCAGCGCGAGTCCACCTTACATCTTGTG
His_6-pp-UbK63R	Ub-K63R QC R	oKMF 074	CACAAGATGTAAGGTGGACTCGCGCTGAATGTTGTAATCGGACAGCGT

Primer sequences are given from the 5'-end to the 3'-end. In the primer name, "F" indicates the forward primer and "R" indicates the reverse primer. Primers shown in red were introduced in ref.[5].

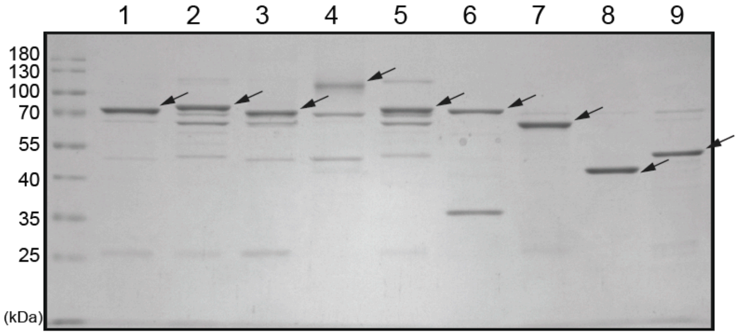

1. Ub$_5$(K48)-GFP-95 (82.3 kDa)
2. 95-GFP-Ub$_5$(K48) (83.2 kDa)
3. Ub$_5$(K63)-GFP-95 (82.3 kDa)
4. Ub$_9$(K63)-GFP-95 (116.5 kDa)
5. 95-GFP-Ub$_5$(K63) (83.0 kDa)
6. Ub$_5$(M1)-GFP-35 (75.4 kDa)
7. Ub$_4$(lin)-GFP-35 (88.5 kDa)
8. UBL-GFP-35 (41.7 kDa)
9. UBL-GFP-95 (48.2 kDa)

Supplementary Figure 1: Ubiquitinated proteasome substrates

Purified ubiquitinated proteasome substrates were analyzed by SDS-PAGE followed by protein staining using Instant Blue. Arrows indicate ubiquitinated substrate. Gel images of Ub$_5$(K48)-GFP-35, Ub$_9$(K48)-GFP-35, Ub$_5$(K63)-GFP-35, Ub$_5$(K11)-GFP-35, 35-GFP-Ub$_5$(K63), Ub-GFP-35, Ub-35-Ub-GFP-35, Ub$_3$(K48)-35-Ub$_3$(K48)-GFP-35 were shown in ref.[5].

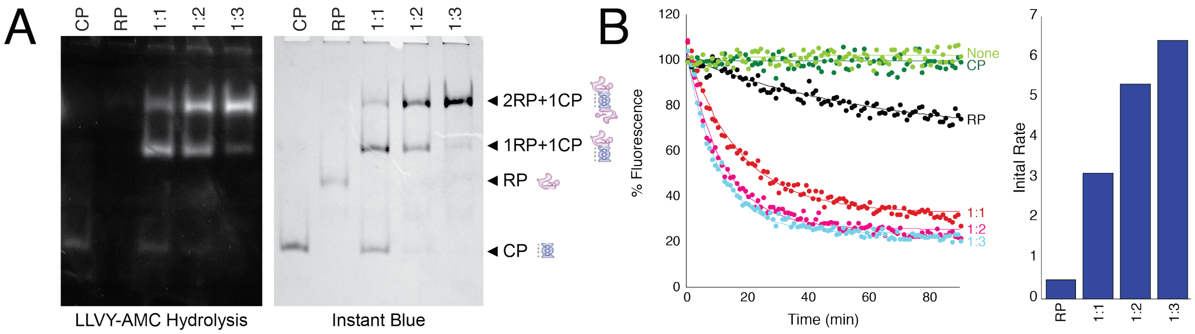

Supplementary Figure 2: Proteasome Reconstitution

(A) Native gel of WT proteasome reconstitution showing in gel hydrolysis of LLVY-AMC (left) or total protein, instant blue staining (right). **(B)** Degradation of Ub_4(lin)-GFP-35 (left) and calculated initial rate (right). Source data are provided as a Source Data file.

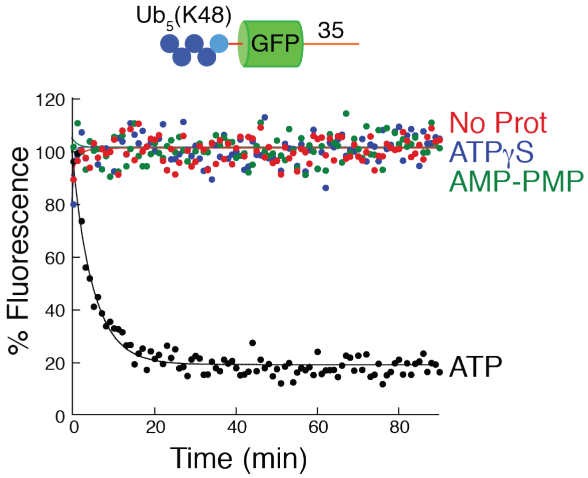

Supplementary Figure 3: ATP Dependence of Proteasome Degradation of Ub_4(K48) Substrate Reaction was of 5 nM substrate with 25 nM CP and 50 nM RP in the presence of 2 mg mL^{-1} BSA, 4 mM DTT, 10 mM Tris-HCl pH 7.5, 1 mM $MgCl_2$, 1% glycerol and 1 mM indicated nucleotide at 30 ˚C. Source data are provided as a Source Data file.

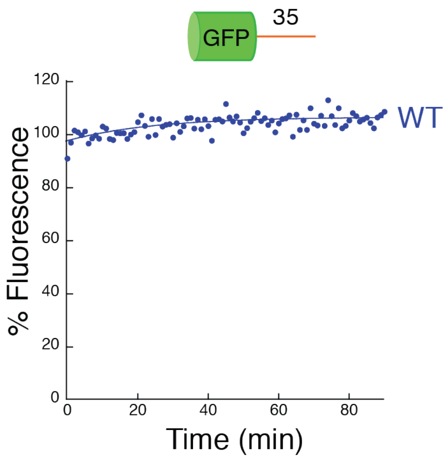

Supplementary Figure 4: Stability of GFP-35 in the Presence of Proteasome

Reaction was of 5 nM substrate with 25 nM CP and 50 nM RP in the presence of 2 mg mL^{-1} BSA, 4 mM DTT, 10 mM Tris-HCl pH 7.5, 1 mM $MgCl_2$, 1% glycerol, 1 mM ATP, 10 mM CP, and 0.1 mg mL^{-1} CPK at 30 ˚C. Degradation was tested together with substrates degraded by the proteasome (not shown). Source data are provided as a Source Data file.

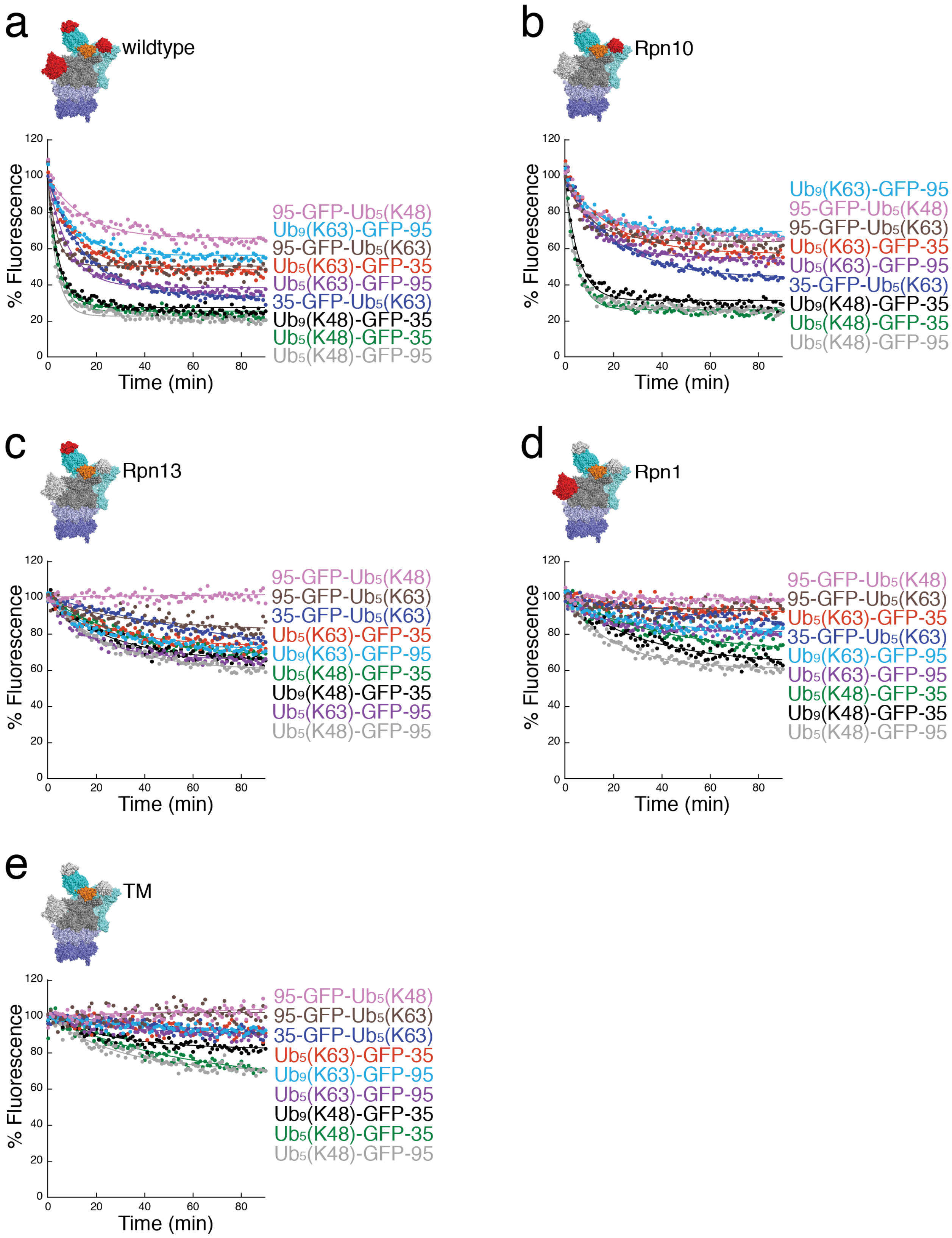

a
wildtype
% Fluorescence
Time (min)
95-GFP-Ub5(K48)
Ub9(K63)-GFP-95
95-GFP-Ub5(K63)
Ub5(K63)-GFP-35
Ub5(K63)-GFP-95
35-GFP-Ub5(K63)
Ub9(K48)-GFP-35
Ub5(K48)-GFP-35
Ub5(K48)-GFP-95
b
Rpn10
% Fluorescence
Time (min)
Ub9(K63)-GFP-95
95-GFP-Ub5(K48)
95-GFP-Ub5(K63)
Ub5(K63)-GFP-35
Ub5(K63)-GFP-95
35-GFP-Ub5(K63)
Ub9(K48)-GFP-35
Ub5(K48)-GFP-35
Ub5(K48)-GFP-95
c
Rpn13
% Fluorescence
Time (min)
95-GFP-Ub5(K48)
95-GFP-Ub5(K63)
35-GFP-Ub5(K63)
Ub5(K63)-GFP-35
Ub9(K63)-GFP-95
Ub5(K48)-GFP-35
Ub9(K48)-GFP-35
Ub5(K63)-GFP-95
Ub5(K48)-GFP-95
d
Rpn1
% Fluorescence
Time (min)
95-GFP-Ub5(K48)
95-GFP-Ub5(K63)
Ub5(K63)-GFP-35
35-GFP-Ub5(K63)
Ub9(K63)-GFP-95
Ub5(K63)-GFP-95
Ub5(K48)-GFP-35
Ub9(K48)-GFP-35
Ub5(K48)-GFP-95
e
TM
% Fluorescence
Time (min)
95-GFP-Ub5(K48)
95-GFP-Ub5(K63)
35-GFP-Ub5(K63)
Ub5(K63)-GFP-35
Ub9(K63)-GFP-95
Ub5(K63)-GFP-95
Ub9(K48)-GFP-35
Ub5(K48)-GFP-35
Ub5(K48)-GFP-95

Supplementary Figure 5: Summary of single turnover degradation reactions by proteasome Figure shows the same data as Figures 2 and 3 but reorganized by proteasome. **(a)** WT proteasome, **(b)** Rpn10 proteasome, **(c)** Rpn13 proteasome, **(d)** Rpn1 proteasome, and **(e)** TM (triple mutant) proteasome. See Supplementary Table 1 for definition of proteasome types.

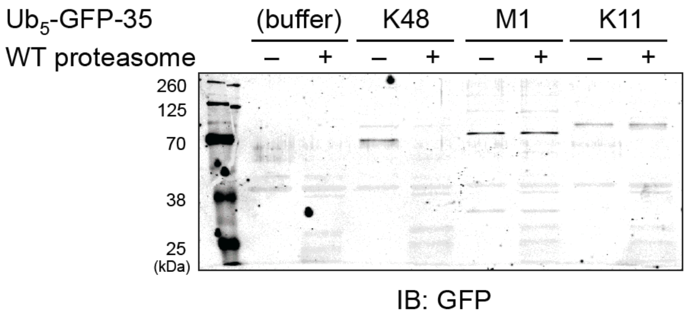

Supplementary Figure 6: Inefficient degradation of substrates modified with M1- and K11-linked ubiquitin chains is not due to deubiquitination

Post reaction mixture of degradation assays of 5 nM of the indicated substrate with or without 25 nM CP and 50 nM RP in the presence of 2 mg mL^{-1} BSA, 4 mM DTT, 10 mM Tris/HCl pH 7.5, 1 mM MgCl2, 1% glycerol, 1 mM ATP, 10 mM CP, and 0.1 mg mL^{-1} CPK at 30 ˚C for 90 min were subjected to SDS-PAGE, followed by immunoblotting with anti GFP antibody (B-2: sc-9996).

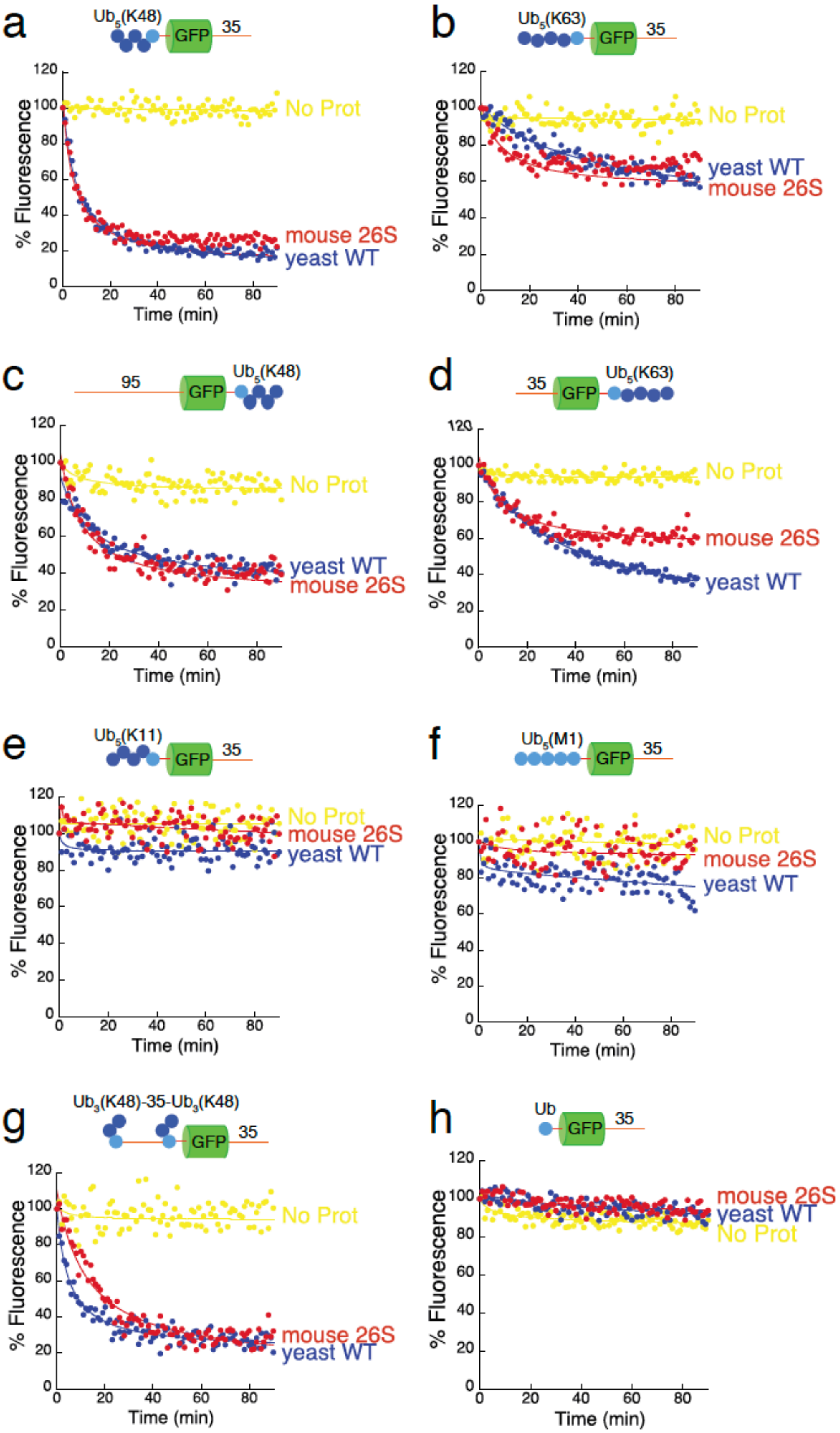
a
Ub5(K48)
GFP
35
No Prot
mouse 26S
yeast WT
% Fluorescence
Time (min)
b
Ub5(K63)
GFP
35
No Prot
yeast WT
mouse 26S
c
95
GFP
Ub5(K48)
No Prot
yeast WT
mouse 26S
d
35
GFP
Ub5(K63)
No Prot
mouse 26S
yeast WT
e
Ub5(K11)
GFP
35
No Prot
mouse 26S
yeast WT
f
Ub5(M1)
GFP
35
No Prot
mouse 26S
yeast WT
g
Ub3(K48)-35-Ub3(K48)
GFP
35
No Prot
mouse 26S
yeast WT
h
Ub
GFP
35
mouse 26S
yeast WT
No Prot

Supplementary Figure 7: Degradation of substrates with ubiquitin chains by yeast and mouse proteasomes

Degradation of 5 nM substrate proteins with the shown ubiquitin chains by yeast wildtype (7.5 nM CP and 15 nM RP) or 7.5 nM mouse 26S proteasome was followed under single turnover conditions in the presence of 1 mM ATP at 37 °C. The graphs show substrate fluorescence as a percentage of the initial fluorescence as a function of time in minutes. Proteasome types are described in Supplementary Table 1. Each panel shows the degradation of substrates as follows: (a) Ub_5(K48)-GFP-35; (b) Ub_5(K63)-GFP-35; (c) 95-GFP-Ub_5(K48); (d) 35-GFP-Ub_5(K63); (e) Ub_5(K11)-GFP-35; (f) Ub_5(M1)-GFP-35; (g) Ub_3(K48)-35-Ub_3(K48)-GFP-35, and (h) Ub-GFP-35. Source data are provided as a Source Data file.

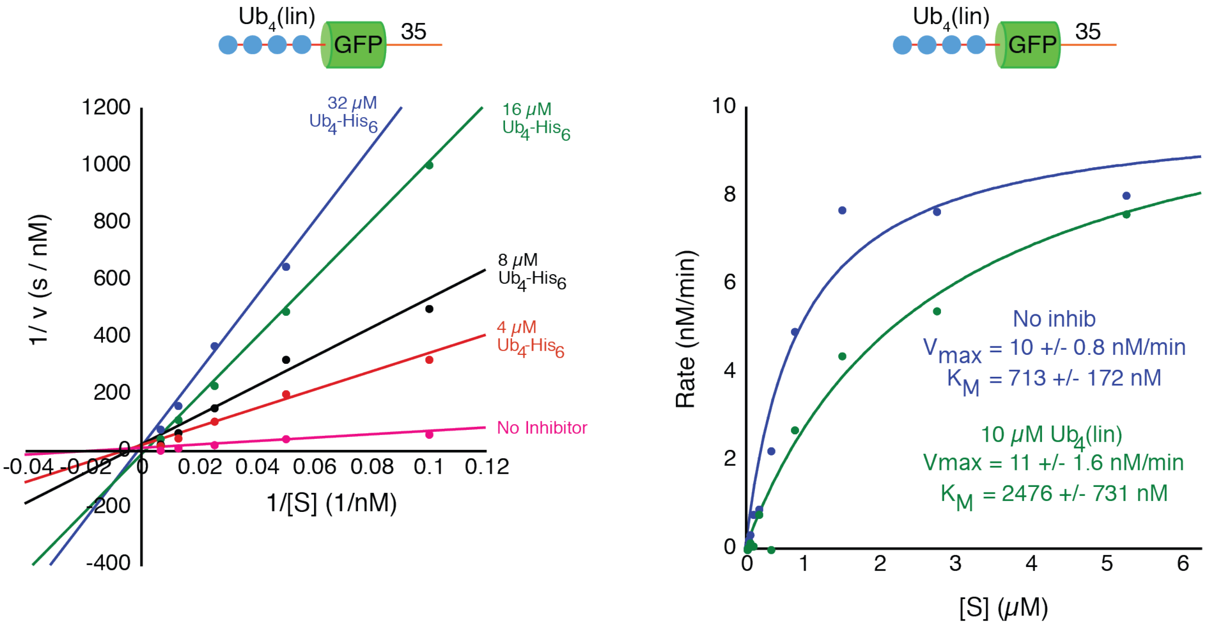

Supplementary Figure 8: Competitive Inhibition Test of Ub_4(lin)-GFP-35 substrate with Ub_4(lin). Source data are provided as a Source Data file.

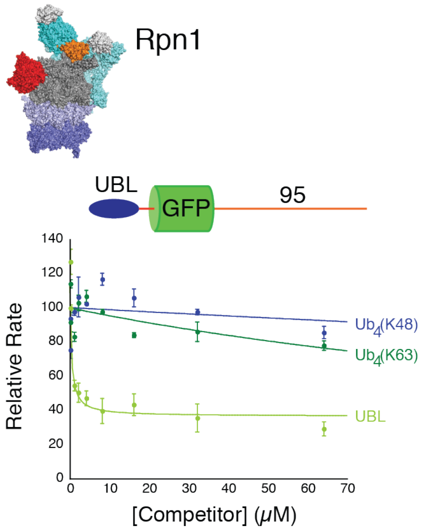

Supplementary Figure 9: Inhibition of UBL-GFP-95 with Indicated Competitors

Degradation of UBL-GFP-95 on Rpn1 proteasome inhibited with various competitors. Increasing concentrations of the indicated competitor were added to single turnover degradation reactions and the initial rate of the reactions determined and fit to determine a K_i. Errors are standard errors from three independent experiments. Source data are provided as a Source Data file.

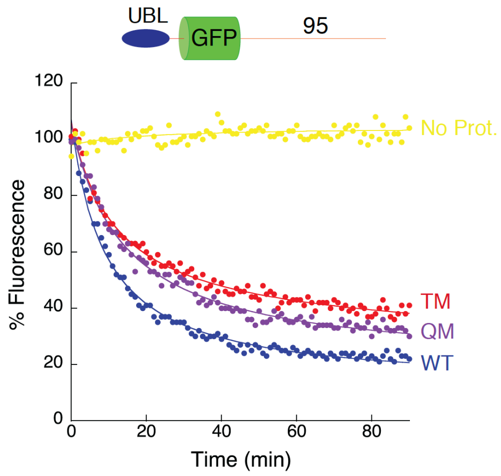

Supplementary Figure 10: Degradation of UBL-GFP-95 by proteasome in which the binding site for the divergent UBL of Ubp6 on Rpn1 has been attenuated

We created QM proteasome by mutating the binding site for the divergent UBL domain of Ubp6 (the T2 binding site) in addition to the UBL binding site on Rpn1 (T1) (i.e., *rpn1-ARR-AKAA* ref.[4]) and the ubiquitin binding sites in Rpn10 and Rpn13. The graph shows degradation of UBL-GFP-95 on WT, TM, and QM proteasome as described. Source data are provided as a Source Data file.

Supplementary Note 2

Coding sequences of ORFs for constructs used in this study:

Legend
Ubiquitin or UBL domain. Cyan and blue if multiple next to one another
Linker
CP8
Initiation region
6x-His tag
Stop codon

1. Ub-CP8-35-His6
ATGGCACAAATCTTCGTGAAGACTTTGACCGGTAAAACCATAACATTGGAAGTT
GAATCTTCCGATACCATCGACAACGTTAAGTCGAAAATTCAAGACAAGGAAGGT
ATCCCTCCAGATCAACAAAGATTGATCTTTGCCGGTAAGCAGCTAGAAGACGGT
AGAACGCTGTCTGATTACAACATTCAGAAGGAGTCCACCTTACATCTTGTGCTA
AGGCTAAGAGGTGTTGGATCCGGTGGTTCGGGCgatggcagcgtgcagctggctgATCA
CTACCAGCAAAACACTCCAATCGGTGATGGTCCTGTTCTGCTGCCAGACAATCA
CTATCTGAGCACGCAAAGCGTTCTGTCTAAAGATCCGAACGAGAAACGCGATCA
TATGGTTCTGCTGGAGTTCGTAACCGCAGCGGGCATCACGCATGGCATGGATG
AACTATACAAAGGTGGTACCGGTGGTTCTATGCGTAAAGGCGAAGAGCTGTTCA
CTGGTGTCGTCCCTATTCTGGTGGAACTGGATGGTGATGTCAACGGTCATAAGT
TTTCCGTGCGTGGCGAGGGTGAAGGTGACGCAACTAATGGTAAACTGACGCTG
AAGTTCATCTGTACTACTGGTAAACTGCCGGTACCTTGGCCGACTCTGGTAACG
ACGCTGACTTATGGTGTTCAGTGCTTTGCTCGTTATCCGGACCATATGAAGCAG
CATGACTTCTTCAAGTCCGCCATGCCGGAAGGCTATGTGCAGGAACGCACGAT
TTCCTTTAAGGATGACGGCACGTACAAAACGCGTGCGGAAGTGAAATTTGAAGG
CGATACCCTGGTAAACCGCATTGAGCTGAAAGGCATTGACTTTAAAGAAGACGG
CAATATCCTGGgCCATAAGCTGGAATACAATTTTAACAGCCACAATGTTTACATC
ACCGCCGATAAACAAAAAAATGGCATTAAAGCGAATTTTAAAATTCGCCACAAC
GTGgAGGGTCCGCGGCTAAGATACCAACCTTTACTAAGAATCTCGCAGAACTGT
GAGGCTGCTATCCTCAGAGCGTCTCAGACTAGATTGAACACGATCAGCGGTAG
GATTTCGCCCGCTGAACACCATCACCATCATCACTAA

2. Ub-CP8-95-His6
ATGGCACAAATCTTCGTGAAGACTTTGACCGGTAAAACCATAACATTGGAAGTT
GAATCTTCCGATACCATCGACAACGTTAAGTCGAAAATTCAAGACAAGGAAGGT
ATCCCTCCAGATCAACAAAGATTGATCTTTGCCGGTAAGCAGCTAGAAGACGGT
AGAACGCTGTCTGATTACAACATTCAGAAGGAGTCCACCTTACATCTTGTGCTA
AGGCTAAGAGGTGTTGGATCCGGTGGTTCGGGCgatggcagcgtgcagctggctgATCA
CTACCAGCAAAACACTCCAATCGGTGATGGTCCTGTTCTGCTGCCAGACAATCA
CTATCTGAGCACGCAAAGCGTTCTGTCTAAAGATCCGAACGAGAAACGCGATCA
TATGGTTCTGCTGGAGTTCGTAACCGCAGCGGGCATCACGCATGGCATGGATG
AACTATACAAAGGTGGTACCGGTGGTTCTATGCGTAAAGGCGAAGAGCTGTTCA
CTGGTGTCGTCCCTATTCTGGTGGAACTGGATGGTGATGTCAACGGTCATAAGT
TTTCCGTGCGTGGCGAGGGTGAAGGTGACGCAACTAATGGTAAACTGACGCTG
AAGTTCATCTGTACTACTGGTAAACTGCCGGTACCTTGGCCGACTCTGGTAACG
ACGCTGACTTATGGTGTTCAGTGCTTTGCTCGTTATCCGGACCATATGAAGCAG
CATGACTTCTTCAAGTCCGCCATGCCGGAAGGCTATGTGCAGGAACGCACGAT
TTCCTTTAAGGATGACGGCACGTACAAAACGCGTGCGGAAGTGAAATTTGAAGG

CGATACCCTGGTAAACCGCATTGAGCTGAAAGGCATTGACTTTAAAGAAGACGG
CAATATCCTGGgCCATAAGCTGGAATACAATTTTAACAGCCACAATGTTTACATC
ACCGCCGATAAACAAAAAAATGGCATTAAAGCGAATTTTAAAATTCGCCACAAC
GTGgAGGGTCCGCGGCTAAGATACCAACCTTTACTAAGAATCTCGCAGAACTGT
GAGGCTGCTATCCTCAGAGCGTCTCAGACTAGATTGAACACGATCGGCGCGTA
CGGTTCTACCGTTCCAAGATCCCAGTCGTTCGAACAAGACTCAAGACAACGCAC
ACAGTCATGGACTGCCTTGAGAGTCGGTGCAATTCCTGCCGCTACTAGTTCCGT
GGCGTATCTAAACTGGCATAATGGCCAAATAGACAACGAGCCGCAACTGGATAT
GAATAGACAAAGGATTTCGCCCGCTGAACACCATCACCATCATCACTAA

3. His6-35-CP8-Ub

ATGGGCAGCAGCCATCACCATCATCACCACAGCCAGGATCCGCTAAGATACCA
ACCTTTACTAAGAATCTCGCAGAACTGTGAGGCTGCTATCCTCAGAGCGTCTCA
GACTAGATTGAACACGATCAGCGGTAGGATTTCGCCCGCTGAAGAATTCgatggc
agcgtgcagctggctgATCACTACCAGCAAAACACTCCAATCGGTGATGGTCCTGTTCT
GCTGCCAGACAATCACTATCTGAGCACGCAAAGCGTTCTGTCTAAAGATCCGAA
CGAGAAACGCGATCATATGGTTCTGCTGGAGTTCGTAACCGCAGCGGGCATCA
CGCATGGCATGGATGAACTATACAAAGGTGGTACCGGTGGTTCTATGCGTAAA
GGCGAAGAGCTGTTCACTGGTGTCGTCCCTATTCTGGTGGAACTGGATGGTGA
TGTCAACGGTCATAAGTTTTCCGTGCGTGGCGAGGGTGAAGGTGACGCAACTA
ATGGTAAACTGACGCTGAAGTTCATCTGTACTACTGGTAAACTGCCGGTACCTT
GGCCGACTCTGGTAACGACGCTGACTTATGGTGTTCAGTGCTTTGCTCGTTATC
CGGACCATATGAAGCAGCATGACTTCTTCAAGTCCGCCATGCCGGAAGGCTAT
GTGCAGGAACGCACGATTTCCTTTAAGGATGACGGCACGTACAAAACGCGTGC
GGAAGTGAAATTTGAAGGCGATACCCTGGTAAACCGCATTGAGCTGAAAGGCA
TTGACTTTAAAGAAGACGGCAATATCCTGGgCCATAAGCTGGAATACAATTTTAA
CAGCCACAATGTTTACATCACCGCCGATAAACAAAAAAATGGCATTAAAGCGAA
TTTTAAAATTCGCCACAACGTGGAGCTCGGATCCGGTGGTTCGGGCATGCAAAT
CTTCGTGAAGACTTTGACCGGTAAAACCATAACATTGGAAGTTGAATCTTCCGAT
ACCATCGACAACGTTAAGTCGAAAATTCAAGACAAGGAAGGTATCCCTCCAGAT
CAACAAAGATTGATCTTTGCCGGTAAGCAGCTAGAAGACGGTAGAACGCTGTCT
GATTACAACATTCAGAAGGAGTCCACCTTACATCTTGTGCTAAGGCTAAGAGGT
GTTTAA

4. His6-95-CP8-Ub

ATGGGCAGCAGCCATCACCATCATCACCACAGCCAGGATCCGCTAAGATACCA
ACCTTTACTAAGAATCTCGCAGAACTGTGAGGCTGCTATCCTCAGAGCGTCTCA
GACTAGATTGAACACGATCGGCGCGTACGGTTCTACCGTTCCAAGATCCCAGT
CGTTCGAACAAGACTCAAGACAACGCACACAGTCATGGACTGCCTTGAGAGTC
GGTGCAATTCCTGCCGCTACTAGTTCCGTGGCGTATCTAAACTGGCATAATGGC
CAAATAGACAACGAGCCGCAACTGGATATGAATAGACAAAGGATTTCGCCCGCT
GAAGAATTCgatggcagcgtgcagctggctgATCACTACCAGCAAAACACTCCAATCGGT
GATGGTCCTGTTCTGCTGCCAGACAATCACTATCTGAGCACGCAAAGCGTTCTG
TCTAAAGATCCGAACGAGAAACGCGATCATATGGTTCTGCTGGAGTTCGTAACC
GCAGCGGGCATCACGCATGGCATGGATGAACTATACAAAGGTGGTACCGGTGG
TTCTATGCGTAAAGGCGAAGAGCTGTTCACTGGTGTCGTCCCTATTCTGGTGGA
ACTGGATGGTGATGTCAACGGTCATAAGTTTTCCGTGCGTGGCGAGGGTGAAG
GTGACGCAACTAATGGTAAACTGACGCTGAAGTTCATCTGTACTACTGGTAAAC
TGCCGGTACCTTGGCCGACTCTGGTAACGACGCTGACTTATGGTGTTCAGTGC
TTTGCTCGTTATCCGGACCATATGAAGCAGCATGACTTCTTCAAGTCCGCCATG
CCGGAAGGCTATGTGCAGGAACGCACGATTTCCTTTAAGGATGACGGCACGTA
CAAAACGCGTGCGGAAGTGAAATTTGAAGGCGATACCCTGGTAAACCGCATTG
AGCTGAAAGGCATTGACTTTAAAGAAGACGGCAATATCCTGGgCCATAAGCTGG

AATACAATTTTAACAGCCACAATGTTTACATCACCGCCGATAAACAAAAAAATGG
CATTAAAGCGAATTTTAAAATTCGCCACAACGTGGAGCTCGGATCCGGTGGTTC
GGGCATGCAAATCTTCGTGAAGACTTTGACCGGTAAAACCATAACATTGGAAGT
TGAATCTTCCGATACCATCGACAACGTTAAGTCGAAAATTCAAGACAAGGAAGG
TATCCCTCCAGATCAACAAAGATTGATCTTTGCCGGTAAGCAGCTAGAAGACGG
TAGAACGCTGTCTGATTACAACATTCAGAAGGAGTCCACCTTACATCTTGTGCTA
AGGCTAAGAGGTGTTTAA

5. Ubi4-CP8-35-His6 (Ub5M1-CP8-35-His6)

ATGCAGATTTTCGTCAAGACTTTGACGGGTAAAACCATAACATTGGAAGTTGAAT
CTTCCGATACCATCGACAACGTTAAGTCGAAAATTCAAGACAAGGAAGGTATCC
CTCCAGATCAACAAAGATTGATCTTTGCCGGTAAGCAGCTAGAAGACGGTAGAA
CGCTGTCTGATTACAACATTCAGAAGGAGTCCACCTTACATCTTGTGCTAAGGC
TAAGAGGTGGTATGCAGATCTTTGTTAAGACTTTGACCGGTAAAACCATCACTTT
AGAGGTTGAATCCTCCGACACGATCGATAACGTTAAGTCGAAAATCCAGGACAA
GGAAGGTATCCCTCCGGATCAACAGAGGTTGATCTTTGCCGGTAAGCAGCTAG
AAGATGGTAGAACCTTGTCTGACTACAACATCCAAAAGGAATCTACTCTTCACTT
GGTGTTGAGACTGAGAGGTGGTATGCAAATTTTTGTCAAGACACTGACAGGTAA
GACTATAACCCTAGAGGTTGAATCTTCTGACACTATCGACAACGTTAAGTCGAA
AATTCAAGACAAGGAAGGTATTCCTCCAGATCAACAAAGATTGATTTTTGCTGGT
AAGCAACTGGAAGACGGTAGAACGCTGTCTGATTATAACATTCAGAAAGAGTCT
ACGTTGCATTTGGTGTTGAGATTGAGAGGTGGTATGCAAATTTTCGTCAAAACT
CTAACAGGGAAGACTATAACCCTAGAGGTTGAATCTTCCGACACTATTGACAAC
GTCAAAAGTAAAATTCAAGATAAAGAAGGTATCCCTCCGGATCAACAGAGATTG
ATTTTTGCTGGTAAGCAACTAGAAGATGGTAGAACCTTGTCTGACTACAACATCC
AAAAGGAATCTACTCTTCACTTGGTGTTGAGACTGAGAGGTGGTATGCAAATTTT
TGTCAAGACACTGACAGGTAAGACTATAACCCTAGAGGTTGAATCTTCTGACAC
TATTGACAACGTTAAGTCGAAAATTCAAGACAAGGAAGGTATTCCTCCAGACCA
GCAAAGATTGATTTTTGCCGGTAAGCAACTAGAAGATGGTAGAACGCTGTCGGA
CTACAATATTCAAAAGGAGTCCACTCTTCACCTTGTCTTGAGGTTGAGGGGTGG
TGGATCCGGTGGTTCGGGCgatggcagcgtgcagctggctgATCACTACCAGCAAAACAC
TCCAATCGGTGATGGTCCTGTTCTGCTGCCAGACAATCACTATCTGAGCACGCA
AAGCGTTCTGTCTAAAGATCCGAACGAGAAACGCGATCATATGGTTCTGCTGGA
GTTCGTAACCGCAGCGGGCATCACGCATGGCATGGATGAACTATACAAAGGTG
GTACCGGTGGTTCTATGCGTAAAGGCGAAGAGCTGTTCACTGGTGTCGTCCCT
ATTCTGGTGGAACTGGATGGTGATGTCAACGGTCATAAGTTTTCCGTGCGTGGC
GAGGGTGAAGGTGACGCAACTAATGGTAAACTGACGCTGAAGTTCATCTGTACT
ACTGGTAAACTGCCGGTACCTTGGCCGACTCTGGTAACGACGCTGACTTATGG
TGTTCAGTGCTTTGCTCGTTATCCGGACCATATGAAGCAGCATGACTTCTTCAA
GTCCGCCATGCCGGAAGGCTATGTGCAGGAACGCACGATTTCCTTTAAGGATG
ACGGCACGTACAAAACGCGTGCGGAAGTGAAATTTGAAGGCGATACCCTGGTA
AACCGCATTGAGCTGAAAGGCATTGACTTTAAAGAAGACGGCAATATCCTGGgC
CATAAGCTGGAATACAATTTTAACAGCCACAATGTTTACATCACCGCCGATAAAC
AAAAAAATGGCATTAAAGCGAATTTTAAAATTCGCCACAACGTGgAGGGTCCGC
GGCTAAGATACCAACCTTTACTAAGAATCTCGCAGAACTGTGAGGCTGCTATCC
TCAGAGCGTCTCAGACTAGATTGAACACGATCAGCGGTAGGATTTCGCCCGCT
GAACACCATCACCATCATCACTAA

6. Ub4-CP8-35-His6 (Ub4linker-CP8-35-His6)

ATGCAAATCTTCGTGAAGACTTTGACCGGTAAAACCATAACATTGGAAGTTGAAT
CTTCCGATACCATCGACAACGTTAAGTCGAAAATTCAAGACAAGGAAGGTATCC
CTCCAGATCAACAAAGATTGATCTTTGCCGGTAAGCAGCTAGAAGACGGTAGAA
CGCTGTCTGATTACAACATTCAGAAGGAGTCCACCTTACATCTTGTGCTAAGGC

TAAGAGGTGTTGGATCTGGTGGTGGCGGCATGCAAATCTTCGTGAAGACTTTGA
CCGGTAAAACCATAACATTGGAAGTTGAATCTTCCGATACCATCGACAACGTTA
AGTCGAAAATTCAAGACAAGGAAGGTATCCCTCCAGATCAACAAAGATTGATCT
TTGCCGGTAAGCAGCTAGAAGACGGTAGAACGCTGTCTGATTACAACATTCAGA
AGGAGTCCACCTTACATCTTGTGCTAAGGCTAAGAGGTGTTGGATCTGGTGGTG
GCGGCATGCAAATCTTCGTGAAGACTTTGACCGGTAAAACCATAACATTGGAAG
TTGAATCTTCCGATACCATCGACAACGTTAAGTCGAAAATTCAAGACAAGGAAG
GTATCCCTCCAGATCAACAAAGATTGATCTTTGCCGGTAAGCAGCTAGAAGACG
GTAGAACGCTGTCTGATTACAACATTCAGAAGGAGTCCACCTTACATCTTGTGC
TAAGGCTAAGAGGTGTTGGATCTGGTGGTGGCGGCATGCAAATCTTCGTGAAG
ACTTTGACCGGTAAAACCATAACATTGGAAGTTGAATCTTCCGATACCATCGACA
ACGTTAAGTCGAAAATTCAAGACAAGGAAGGTATCCCTCCAGATCAACAAAGAT
TGATCTTTGCCGGTAAGCAGCTAGAAGACGGTAGAACGCTGTCTGATTACAACA
TTCAGAAGGAGTCCACCTTACATCTTGTGCTAAGGCTAAGAGGTGTTGGATCCG
GTGGTTCGGGCgatggcagcgtgcagctggctgATCACTACCAGCAAAACACTCCAATCG
GTGATGGTCCTGTTCTGCTGCCAGACAATCACTATCTGAGCACGCAAAGCGTTC
TGTCTAAAGATCCGAACGAGAAACGCGATCATATGGTTCTGCTGGAGTTCGTAA
CCGCAGCGGGCATCACGCATGGCATGGATGAACTATACAAAGGTGGTACCGGT
GGTTCTATGCGTAAAGGCGAAGAGCTGTTCACTGGTGTCGTCCCTATTCTGGTG
GAACTGGATGGTGATGTCAACGGTCATAAGTTTTCCGTGCGTGGCGAGGGTGA
AGGTGACGCAACTAATGGTAAACTGACGCTGAAGTTCATCTGTACTACTGGTAA
ACTGCCGGTACCTTGGCCGACTCTGGTAACGACGCTGACTTATGGTGTTCAGT
GCTTTGCTCGTTATCCGGACCATATGAAGCAGCATGACTTCTTCAAGTCCGCCA
TGCCGGAAGGCTATGTGCAGGAACGCACGATTTCCTTTAAGGATGACGGCACG
TACAAAACGCGTGCGGAAGTGAAATTTGAAGGCGATACCCTGGTAAACCGCATT
GAGCTGAAAGGCATTGACTTTAAAGAAGACGGCAATATCCTGGgCCATAAGCTG
GAATACAATTTTAACAGCCACAATGTTTACATCACCGCCGATAAACAAAAAAATG
GCATTAAAGCGAATTTTAAAATTCGCCACAACGTGgAGGGTCCGCGGCTAAGAT
ACCAACCTTTACTAAGAATCTCGCAGAACTGTGAGGCTGCTATCCTCAGAGCGT
CTCAGACTAGATTGAACACGATCAGCGGTAGGATTTCGCCCGCTGAACACCATC
ACCATCATCACTAA

7. Ub-35-Ub-CP8-35-His6

ATGCAAATCTTCGTTAAAACCCTGACCGGTAAAACCATCACCCTGGAAGTTGAA
TCTTCTGACACCATCGACAACGTTAAATCTAAAATCCAGGACAAAGAAGGTATC
CCGCCGGACCAGCAGCGTCTGATCTTCGCTGGTAAACAGCTGGAAGACGGTCG
TACCCTGTCTGACTACAACATCCAGAAAGAATCTACCCTGCACCTGGTTCTGCG
TCTGCGTGGTGTTAGATACCAACCTTTACTAAGAATCTCGCAGAACTGTGAGGC
TGCTATCCTCAGAGCGTCTCAGACTAGATTGAACACGATCAGCGGTAGGATTTC
GCCCGCTGAAATGCAGATATTTGTGAAGACTTTGACCGGTAAAACCATAACATT
GGAAGTTGAATCTTCCGATACCATCGACAACGTTAAGTCGAAAATTCAAGACAA
GGAAGGTATCCCTCCAGATCAACAAAGATTGATCTTTGCCGGTAAGCAGCTAGA
AGACGGTAGAACGCTGTCTGATTACAACATTCAGAAGGAGTCCACCTTACATCT
TGTGCTAAGGCTAAGAGGTGTTGGATCCGGTGGTTCGGGCgatggcagcgtgcagctg
gctgATCACTACCAGCAAAACACTCCAATCGGTGATGGTCCTGTTCTGCTGCCAG
ACAATCACTATCTGAGCACGCAAAGCGTTCTGTCTAAAGATCCGAACGAGAAAC
GCGATCATATGGTTCTGCTGGAGTTCGTAACCGCAGCGGGCATCACGCATGGC
ATGGATGAACTATACAAAGGTGGTACCGGTGGTTCTATGCGTAAAGGCGAAGA
GCTGTTCACTGGTGTCGTCCCTATTCTGGTGGAACTGGATGGTGATGTCAACG
GTCATAAGTTTTCCGTGCGTGGCGAGGGTGAAGGTGACGCAACTAATGGTAAA
CTGACGCTGAAGTTCATCTGTACTACTGGTAAACTGCCGGTACCTTGGCCGACT
CTGGTAACGACGCTGACTTATGGTGTTCAGTGCTTTGCTCGTTATCCGGACCAT

ATGAAGCAGCATGACTTCTTCAAGTCCGCCATGCCGGAAGGCTATGTGCAGGA
ACGCACGATTTCCTTTAAGGATGACGGCACGTACAAAACGCGTGCGGAAGTGA
AATTTGAAGGCGATACCCTGGTAAACCGCATTGAGCTGAAAGGCATTGACTTTA
AAGAAGACGGCAATATCCTGGgCCATAAGCTGGAATACAATTTTAACAGCCACA
ATGTTTACATCACCGCCGATAAACAAAAAAATGGCATTAAAGCGAATTTTAAAAT
TCGCCACAACGTGgAGGGTCCGCGGCTAAGATACCAACCTTTACTAAGAATCTC
GCAGAACTGTGAGGCTGCTATCCTCAGAGCGTCTCAGACTAGATTGAACACGAT
CAGCGGTAGGATTTCGCCCGCTGAACACCATCACCATCATCACTAA

8. UBL(80)-CP8-35-His6

ATGGTTAGCTTAACCTTTAAAAATTTCAAGAAGGAAAAAGTTCCTTTAGATCTGG
AACCTTCAAACACAATTTTAGAGACCAAGACCAAGCTTGCTCAATCCATTTCTTG
TGAAGAATCTCAAATAAAACTGATCTACTCGGGTAAAGTGCTACAAGATTCCAAA
ACCGTATCGGAATGCGGGCTAAAAGATGGGGACCAAGTTGTCTTCATGGTTTCT
CAAAAAAAGTCCACGAAGACCGGATCCGGTGGTTCGGGCgatggcagcgtgcagctggc
tgATCACTACCAGCAAAACACTCCAATCGGTGATGGTCCTGTTCTGCTGCCAGAC
AATCACTATCTGAGCACGCAAAGCGTTCTGTCTAAAGATCCGAACGAGAAACGC
GATCATATGGTTCTGCTGGAGTTCGTAACCGCAGCGGGCATCACGCATGGCAT
GGATGAACTATACAAAGGTGGTACCGGTGGTTCTATGCGTAAAGGCGAAGAGC
TGTTCACTGGTGTCGTCCCTATTCTGGTGGAACTGGATGGTGATGTCAACGGTC
ATAAGTTTTCCGTGCGTGGCGAGGGTGAAGGTGACGCAACTAATGGTAAACTG
ACGCTGAAGTTCATCTGTACTACTGGTAAACTGCCGGTACCTTGGCCGACTCTG
GTAACGACGCTGACTTATGGTGTTCAGTGCTTTGCTCGTTATCCGGACCATATG
AAGCAGCATGACTTCTTCAAGTCCGCCATGCCGGAAGGCTATGTGCAGGAACG
CACGATTTCCTTTAAGGATGACGGCACGTACAAAACGCGTGCGGAAGTGAAATT
TGAAGGCGATACCCTGGTAAACCGCATTGAGCTGAAAGGCATTGACTTTAAAGA
AGACGGCAATATCCTGGgCCATAAGCTGGAATACAATTTTAACAGCCACAATGTT
TACATCACCGCCGATAAACAAAAAAATGGCATTAAAGCGAATTTTAAAATTCGCC
ACAACGTGgAGGGTCCGCGGCTAAGATACCAACCTTTACTAAGAATCTCGCAGA
ACTGTGAGGCTGCTATCCTCAGAGCGTCTCAGACTAGATTGAACACGATCAGC
GGTAGGATTTCGCCCGCTGAACACCATCACCATCATCACTAA

9. UBL(80)-CP8-95-His6

ATGGTTAGCTTAACCTTTAAAAATTTCAAGAAGGAAAAAGTTCCTTTAGATCTGG
AACCTTCAAACACAATTTTAGAGACCAAGACCAAGCTTGCTCAATCCATTTCTTG
TGAAGAATCTCAAATAAAACTGATCTACTCGGGTAAAGTGCTACAAGATTCCAAA
ACCGTATCGGAATGCGGGCTAAAAGATGGGGACCAAGTTGTCTTCATGGTTTCT
CAAAAAAAGTCCACGAAGACCGGATCCGGTGGTTCGGGCgatggcagcgtgcagctggc
tgATCACTACCAGCAAAACACTCCAATCGGTGATGGTCCTGTTCTGCTGCCAGAC
AATCACTATCTGAGCACGCAAAGCGTTCTGTCTAAAGATCCGAACGAGAAACGC
GATCATATGGTTCTGCTGGAGTTCGTAACCGCAGCGGGCATCACGCATGGCAT
GGATGAACTATACAAAGGTGGTACCGGTGGTTCTATGCGTAAAGGCGAAGAGC
TGTTCACTGGTGTCGTCCCTATTCTGGTGGAACTGGATGGTGATGTCAACGGTC
ATAAGTTTTCCGTGCGTGGCGAGGGTGAAGGTGACGCAACTAATGGTAAACTG
ACGCTGAAGTTCATCTGTACTACTGGTAAACTGCCGGTACCTTGGCCGACTCTG
GTAACGACGCTGACTTATGGTGTTCAGTGCTTTGCTCGTTATCCGGACCATATG
AAGCAGCATGACTTCTTCAAGTCCGCCATGCCGGAAGGCTATGTGCAGGAACG
CACGATTTCCTTTAAGGATGACGGCACGTACAAAACGCGTGCGGAAGTGAAATT
TGAAGGCGATACCCTGGTAAACCGCATTGAGCTGAAAGGCATTGACTTTAAAGA
AGACGGCAATATCCTGGgCCATAAGCTGGAATACAATTTTAACAGCCACAATGTT
TACATCACCGCCGATAAACAAAAAAATGGCATTAAAGCGAATTTTAAAATTCGCC
ACAACGTGgAGGGTCCGCGGCTAAGATACCAACCTTTACTAAGAATCTCGCAGA
ACTGTGAGGCTGCTATCCTCAGAGCGTCTCAGACTAGATTGAACACGATCGGC

GCGTACGGTTCTACCGTTCCAAGATCCCAGTCGTTCGAACAAGACTCAAGACAA
CGCACACAGTCATGGACTGCCTTGAGAGTCGGTGCAATTCCTGCCGCTACTAG
TTCCGTGGCGTATCTAAACTGGCATAATGGCCAAATAGACAACGAGCCGCAACT
GGATATGAATAGACAAAGGATTTCGCCCGCTGAACACCATCACCATCATCACTA
A

Supplementary References

1. Sone T, Saeki Y, Toh-e A, Yokosawa H. Sem1p is a novel subunit of the 26 S proteasome from Saccharomyces cerevisiae. *J Biol Chem* **279**, 28807-28816 (2004).

2. Finley D, Ozkaynak E, Varshavsky A. The yeast polyubiquitin gene is essential for resistance to high temperatures, starvation, and other stresses. *Cell* **48**, 1035-1046 (1987).

3. Fu H*, et al.* Structure and functional analysis of the 26S proteasome subunits from plants. *Mol Biol Rep* **26**, 137-146 (1999).

4. Shi Y*, et al.* Rpn1 provides adjacent receptor sites for substrate binding and deubiquitination by the proteasome. *Science* **351**, aad9421 (2016).

5. Martinez-Fonts K, Matouschek A. A Rapid and Versatile Method for Generating Proteins with Defined Ubiquitin Chains. *Biochemistry* **55**, 1898-1908 (2016).
